# Bridging Medical Specialities in the Management of Polycystic Ovary Syndrome: Integrating Lessons from Sodium–glucose Cotransporter-2 Inhibitors into a Holistic Approach

**DOI:** 10.17925/EE.2025.21.2.1

**Published:** 2025-04-07

**Authors:** Taieb Ach, Feryel Amri, Houcem ElOmma Mrabet

**Affiliations:** 1. Faculty of Medicine of Sousse, University of Sousse, Sousse, Tunisia; 2. Department of Endocrinology, University Hospital of Farhat Hached Sousse, Sousse, Tunisia; 3. Laboratory of Exercise Physiology and Pathophysiology (LR19ES09), Sousse, Tunisia; 4. Department of Dermatology, University Hospital of La Rabta Tunis, Tunis, Tunisia; 5. Department of Endocrinology, University Tahar Sfar La Mahdia, Mahdia, Tunisia

**Keywords:** Dermatology, endocrinology, gynaecology, insulin resistance, metabolism, polycystic ovary syndrome (PCOS)

## Abstract

Polycystic ovary syndrome presents significant metabolic, dermatologic and gynaecologic challenges, including hyperandrogenism, menstrual irregularities and obesity. While patients often consult different specialists for dermatologic or gynaecologic concerns, effective management requires placing metabolic health at the centre of all specialities involved; from endocrinology to nutrition. Traditional boundaries between specialities are fading, creating a more unified approach focused on metabolic management. This centralization fosters comprehensive strategies to address both immediate symptoms and long-term risks, leading to improved, holistic patient outcomes.

Polycystic ovary syndrome (PCOS) is a complex, multisystemic condition characterized by reproductive, metabolic and dermatologic manifestations, including hyperandrogenism and ovulatory dysfunction. Despite its prevalence and significant impact on quality of life, PCOS remains underdiagnosed and poorly managed due to its heterogeneous presentation and the lack of a unified approach to care. PCOS affects approximately 6–20% of women of reproductive age globally, representing one of the most common yet complex endocrine disorders.^1^ While polycystic ovaries have traditionally been treated as a gynaecological disorder, it is now recognized that PCOS is a multifaceted condition with systemic implications affecting metabolism, reproductive health and dermatologic presentation.^2^ The syndrome is typically defined by three core diagnostic criteria: clinical and/or biological hyperandrogenism, ovulatory dysfunction and polycystic ovarian morphology (or elevated anti-Müllerian hormone [AMH] levels as an alternative to ovarian morphology assessment), with at least two criteria required for diagnosis.^2^ However, PCOS varies significantly in its clinical presentation, challenging the conventional boundaries between medical specialities and urging a more integrated approach to diagnosis and treatment.^1^

PCOS presents a unique pathophysiological profile that combines hormonal imbalances with metabolic disturbances, particularly insulin resistance and chronic low-grade inflammation, which contribute to its diverse clinical manifestations.^3^ This constellation of symptoms frequently leads patients to seek care from various specialists, frequently without an initial diagnosis of PCOS, due to the singular nature of their complaints – whether menstrual irregularities, infertility, hirsutism, acne or weight gain.^4^ Consequently, it has become crucial for healthcare providers across specialities to recognize the broad spectrum of PCOS symptoms and potential systemic consequences, including type 2 diabetes (T2D), cardiovascular disease and psychological comorbidities.^5^

## Explaining the complex pathophysiology of polycystic ovary syndrome

PCOS is a multifactorial condition driven by a complex interplay of genetic, hormonal, metabolic and environmental factors.^3^ These interactions result in a self-perpetuating cycle of dysfunction that manifests as hyperandrogenism, insulin resistance and ovarian dysfunction, which are central to the syndrome’s pathophysiology.^6^ This intricate web of dysfunction leads to symptoms that can vary widely among patients, often resulting in misdiagnosis or delayed treatment due to a lack of awareness about the condition’s multifaceted nature.^4^

Genetic predisposition, epigenetic modifications and environmental factors, such as diet and lifestyle, play significant roles in shaping the pathophysiology of PCOS.^4^ Elevated androgen levels, primarily due to excessive ovarian androgen production by theca cells, are a hallmark of PCOS.^4^ This is exacerbated by increased luteinizing hormone (LH) secretion from the pituitary gland and amplified by insulin resistance, which further stimulates ovarian androgen synthesis. Hyperandrogenism contributes to clinical features such as hirsutism, acne and alopecia, as well as ovulatory dysfunction. Approximately 60% of women with PCOs are hirsute and have acne, which are the most common clinical signs of hyperandrogenaemia.^7^ This has been evident on clinical examination in a substantial percentage of obese women with PCOS as well as in some lean-affected women. Its severity is directly correlated with the degree of insulin resistance. Additionally, many studies have confirmed a high prevalence of non-alcoholic fatty liver disease in women with PCOS.^8^

Insulin resistance, present in approximately 50–80% of women with PCOS, plays a pivotal role in the syndrome’s metabolic and reproductive disturbances.^6^ It is more prevalent in individuals with overweight or obesity and those meeting the stricter diagnostic criteria set by the National Institutes of Health.^5^ Insulin resistance exacerbates hyperandrogenism by increasing ovarian androgen production and reducing sex hormone-binding globulin levels, leading to higher free androgen levels. Additionally, insulin resistance contributes to metabolic complications such as dyslipidaemia, T2D and cardiovascular risk. Women with PCOS are at increased risk of developing T2D, with a prevalence rate of 7.5% compared to 0% for T2D in age-matched and weight-matched non-PCOS control women.^8^

Ovarian dysfunction in PCOS is characterized by follicular arrest and anovulation, which is driven by hormonal imbalances, including elevated LH and AMH levels.^6^ These disruptions lead to the formation of polycystic ovarian morphology and contribute to infertility. Moreover, chronic low-grade inflammation and oxidative stress further exacerbate the metabolic and reproductive abnormalities in PCOS, creating a vicious cycle that perpetuates the syndrome.

Collaboration among endocrinologists, gynaecologists, nutritionists and dermatologists is essential to unravel the complexities of PCOS, enabling a comprehensive evaluation and tailored management strategies that address both immediate symptoms and long-term health risks. Such integration ensures that all aspects of the syndrome are considered, ultimately improving patient outcomes and reducing the burden of this prevalent disorder.

## Enhancing polycystic ovary syndrome care through cardiorenal perspectives: Lessons from gliflozins’ impact

Initially developed to reduce blood glucose levels in T2D, gliflozin (sodium–glucose cotransporter-2 [SGLT2] inhibitors) revealed an unexpected but beneficial impact on cardiovascular and renal outcomes. Its effects on lowering cardiovascular risk and preserving renal function in patients with diabetes have emphasized the need for a combined cardiometabolic approach.^9^ The advent of gliflozins has effectively unified the management of cardiovascular, T2D and renal risks, fostering collaboration among cardiologists, nephrologists and endocrinologists.

This medication has facilitated a holistic approach to cardiorenal syndrome, where all specialists work together to address the interconnected risks without remaining isolated in their respective domains.^10^ Cardiologists no longer focus solely on cardiovascular risk, nephrologists are not solely concerned with renal function and endocrinologists integrate diabetes management seamlessly into the overall care strategy. This collaborative effort ensures that both cardiovascular and renal health are prioritized, ultimately enhancing patient outcomes through comprehensive, multidisciplinary care.

This model underscores a significant shift towards interdisciplinary patient management, where cardiologists, endocrinologists, nephrologists and nutritionists work together to optimize treatment outcomes. This collaboration, prompted by gliflozin’s wide-reaching effects, serves as a valuable framework for managing other complex, multisystemic conditions.

PCOS, similar to cardiorenal syndrome, blurs the lines between medical specialities due to its multisystemic nature, often leading patients to consult various specialists based on isolated symptoms. This fragmentation can complicate diagnosis and result in disjointed care.^11^

A unified approach is essential for effective PCOS management, whereby specialists no longer focus solely on the symptoms relevant to their specific domain. Instead, centralizing care around metabolic health allows for the simultaneous treatment of reproductive issues by gynaecologists, metabolic complications such as insulin resistance and obesity by endocrinologists, skin symptoms by dermatologists and mental health concerns by mental health specialists. By collaborating in this manner, healthcare providers can develop comprehensive treatment plans that address the interconnected symptoms and systemic complications of PCOS, ensuring holistic and effective patient care.

Just as gliflozins have revolutionized the management of cardiorenal and metabolic risks by fostering collaboration among specialists, a similar approach can transform PCOS care. By breaking down the barriers between specialities and focusing on the patient as a whole, we can address the root causes of PCOS and improve outcomes across all its manifestations. This integrated model highlights the importance of viewing PCOS not as a collection of isolated symptoms but as a systemic condition requiring coordinated, multidisciplinary care.

## Cross-symptom benefits of targeted treatments

A multidisciplinary approach to PCOS management allows each specialist to prescribe treatments that exert pleiotropic effects on the condition. Combined oral contraceptives can be prescribed by gynaecologists to improve menstrual regularity and lower androgen levels, effectively addressing both reproductive issues and dermatologic symptoms such as acne and hirsutism.^2^ Metformin, primarily an insulin-sensitizing agent, can be used by endocrinologists and other specialists to enhance insulin sensitivity, improve glucose tolerance and support ovulation.^12^ Its ability to address metabolic, reproductive and even dermatologic concerns underscores its importance in comprehensive PCOS care.

Lifestyle modifications are fundamental to effective PCOS management. Implementing a hypocaloric diet and encouraging regular physical activity should be emphasized by all specialists involved in a patient’s care. Calorie restriction is as important as food composition, and Shang et al. revealed that calorie-restricted diets may be the optimal choices for reducing insulin resistance and improving body composition.^13^ Interestingly, even though differences in diet structure can have different effects on PCOS theoretically, studies show that weight loss is most beneficial for patients with obesity, regardless of the composition of the diet.^11^

Emerging evidence also highlights the potential role of SGLT2 inhibitors in managing PCOS, particularly through their effects on insulin sensitivity, androgen levels and menstrual regularity. Studies suggest that SGLT2 inhibitors may offer metabolic benefits similar to metformin, along with the added advantage of potential cardioprotective effects.^14^ However, their impact on hyperandrogenism and menstrual regulation remains inconsistent across studies, likely due to variations in drug type, dosage, duration and patient characteristics such as baseline insulin resistance and body mass index. Despite these inconsistencies, SGLT2 inhibitors represent a promising addition to the therapeutic options available for PCOS management, particularly for patients with concurrent cardiometabolic risks. Further research is needed to fully understand their role and establish clear guidelines for their use in this patient population.

By recognizing the interconnectedness of PCOS symptoms and the pleiotropic nature of these treatments, all healthcare providers can collaborate to optimize management strategies, ultimately leading to improved patient outcomes. This integrated approach, inspired by advancements in therapies such as gliflozins, underscores the importance of addressing PCOS as a systemic condition requiring coordinated, multidisciplinary care.

## Conclusions

PCOS is the most common endocrine disorder in women at reproductive age, associated with a plethora of cardiometabolic consequences, with obesity, insulin resistance and hyperandrogenaemia playing a major role in the degree of such manifestations.^1^ These consequences include increased risk of glucose intolerance and TD2M, dyslipidaemia, systemic inflammation, fatty liver disease and hypertension.

PCOS exemplifies a condition that transcends traditional medical boundaries, demanding a multidisciplinary approach to effectively address its multifactorial nature and interconnected symptoms. By fostering collaboration among gynaecologists, endocrinologists, dermatologists, mental health specialists and others, the healthcare system can provide more comprehensive care that aligns with the complex pathophysiology of PCOS. Each specialist can prescribe treatments such as combined oral contraceptives, metformin and lifestyle modifications that not only target specific symptoms but also exert pleiotropic effects, enhancing overall management. Future advancements in PCOS management should continue to prioritize interdisciplinary treatment models, as well as research into therapies that target its systemic underpinnings. This approach not only optimizes symptom control but also addresses the long-term risks associated with PCOS, ultimately improving patient outcomes and quality of life.
